# Hyphenation of Trapped Ion Mobility to Two-Dimensional Mass Spectrometry for Protein Analysis in Complex Biomixtures

**DOI:** 10.1021/jasms.5c00292

**Published:** 2025-11-18

**Authors:** Callan Littlejohn, Meng Li, Christopher A. Wootton, Mark P. Barrow, Peter B. O’Connor

**Affiliations:** † Department of Chemistry, 2707 University of Warwick, Coventry CV4 7AL, U.K.; ‡ Advanced Mass Spectrometry RTP, 2707 University of Warwick, Coventry CV4 7AL, U.K.; § 39117 Bruker Daltonics GmbH & Co. Kg, Bremen 28359, Germany

## Abstract

The analysis of complex biological mixtures remains a significant challenge in mass spectrometry (MS), particularly when using conventional direct infusion MS/MS approaches due to inherent limitations in resolving power and spectral complexity. Here, we demonstrate the integration of trapped ion mobility spectrometry (TIMS) with two-dimensional mass spectrometry (2DMS) to enable high-resolution TIMS-MS/2DMS experiments for detailed protein characterization within mixtures. TIMS provides separation based on the ion’s size-to-charge ratio, effectively reducing the occurrence of chimeric tandem mass spectra containing fragments from more than one precursor ion. This coupling allows for an improved peak capacity and reduced ambiguity in tandem spectral interpretation. When applied to a model protein mixture, the TIMS-MS/2DMS method allows resolution of near *m*/*z* species, including isomeric and isonucleonic species, and it was possible to assign secondary fragmentation with greater confidence.

## Graphical abstract

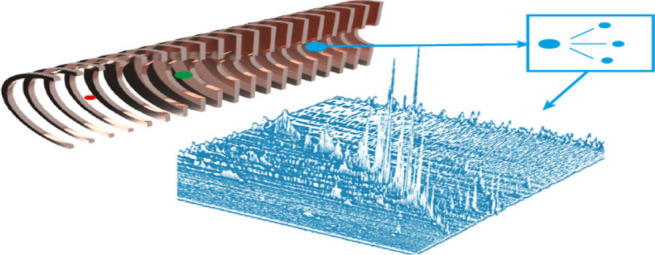

## Introduction

Ion mobility was initially discovered in the early 1900s^1^ and was developed into modern ion mobility spectrometry (IMS) by Earl McDaniel in the 1950s and 1960s,^1–4^ where ions are separated based on their mobility, a parameter determined by an applied electric field, the charge, and the collisional cross sectional area of ions. In IMS, force is imparted on ions using electric fields and a background gas; the larger the ions, the higher the likelihood of collision with the background gas, and therefore, the force imparted by the gas will be higher.

The first IMS experiments involved a drift tube, where ions were accelerated through a stationary background gas, meaning that physically larger ions will experience more resisting force from the background gas and are therefore eluted from the drift tube later. Drift tube IMS was successful in separating ions of different sizes, but its resolving power relied heavily on the length of the drift tube. The size of drift tubes limited its adoption in commercial instruments, and ion mobility spectrometry remained mostly on home-built instrumentation. The velocity of an analyte through the background gas can be defined by

$$v_{d}=qKE$$ 1

where *q* is the charge on the molecule, *v*_d_ is the velocity of the analyte through the gas, *K* is a mobility constant influenced by ion size and gas properties, and *E* is the electric field strength.^5^ It is possible to consider the force exerted by the gas as a frictional force which directly opposes the effect of any electrical field.^6^ The ions experience many opportunities for collision along the path, and therefore, a statistical distribution is observed in the final spectrum with a peak centering on the ion mobility which is a measure of the frictional force from the gas collisions.

Drift tube IMS devices tended to be large, as increasing the length would generally increase resolution. The length of the tube limited its adoption in commercial systems; more modern methods of ion mobility were developed after the drift tube, such as traveling wave ion mobility (TWIMS), structures for lossless ion manipulations (SLIM),^7–13^ and fast switching asymmetric field ion mobility (FAIMS).^14–19^ Additionally, trapped ion mobility spectrometry has recently been introduced allowing for high resolution in mobility separations^17,20–22^ in a very compact geometry.^16,18–31,18–20,22–33^ The principle of operation for TIMS is an opposing electric field to the ion current with a gas flow in the direction of the ion current. The frictional force of the gas flow on the ion will perfectly match the repulsive force of the electric field on the charge at a given point, at which the ion will be essentially “parked” or trapped in place. Due to the inverse-to-typical direction of the electric field, ions in TIMS elute in decreasing size order described using the following equations:^16,19^

$$\mathrm{ccs}=\frac{3q}{16N_{0}}\times \sqrt{\frac{2\pi }{\mu k_{b}T}}\times \frac{1}{K_{0}}$$ 2

$$K_{0}=\frac{3q}{16N_{0}}\times \sqrt{\frac{2\pi }{\mu k_{b}T}}\times \frac{1}{\mathrm{ccs}}$$ 3

$$N_{0}=\frac{P_{0}}{k_{b}T_{0}}$$ 4

$$\mu =\frac{m_{\mathrm{ion}}m_{\mathrm{gas}}}{m_{\mathrm{ion}}+m_{\mathrm{gas}}}$$ 5

where *z* is the charge on the ion *e* is the elementary charge constant; *N*_0_ is the reduced gas number density, which is a constant defined by eq 4, where *P*_0_ is standard pressure and *T*_0_ is standard temperature; μ is the reduced mass defined by eq 5, where *m*_ion_ is the mass of the ion and *m*_gas_ is the mass of the carrier gas; *k*_b_ is the Boltzmann constant; *T* is the temperature of the system; *K*_0_ is a mobility constant determined by the ion size and carrier gas properties; and ccs is the collisional cross-sectional area. The resolution in TIMS has been defined as

$$R=v_{g}\times \sqrt[{4}]{\frac{2L_{P}}{\beta }}\times \frac{1}{\sqrt[{4}]{{K_{0}}^{3}}}\times \sqrt{\frac{q}{16\ln\left(2\right)k_{b}T}}$$ 6

where *v*_g_ is the carrier gas velocity, *L*_p_ is the length of the separation region, β is the rate of change of the electric field, *q* is the charge of the ion, *k*_b_ is the Boltzmann constant, and *T* is the temperature of the system.^34^ The relationship of resolution to length of the drift tube, if all other variables are maintained constant, is shown in eq 7:

$$R\propto \sqrt[{4}]{L_{P}}$$ 7

This means that the effect of the length of the separation zone has very little effect on the final resolving power, which means that TIMS devices can have a considerably smaller footprint than their drift tube counterparts.

TIMS has also been presented in a gated capacity when coupled to a Fourier transform ion cyclotron resonance (FTICR) mass spectrometer.^35^ In “gated TIMS”,^35,36^ only a select region of ion mobilities are allowed to continue to the spectrometer by applying potential differences such that any ions which elute before or after the set gate will be ejected from the main ion beam. Gated TIMS allows for the analysis to take place on an FTICR MS as the detection time of an FTICR MS is relatively long, and thus filtering in this way allows for a transient to be acquired for each scan. The selected analytes of interest proceeded to the rest of the spectrometer. Due to the high degree of reproducibility of gated TIMS, it can be used for MS/MS where ions are first isolated through their mobilities and then fragmented and analyzed. The technique has recently been applied for the characterization of biomixtures^37^ and has the potential to separate near *m*/*z* species which could not be separable by conventional *m*/*z*-based methods such as quadrupole isolation. The high resolving power of the trapped ion mobility device creates a possibility for a massive increase in peak capacity of a data set, which would greatly increase the capability for analysis of more complex mixtures.^38^

Another high peak capacity technique in FTICR-MS is two-dimensional mass spectrometry (2DMS).^39–41^ 2DMS is a true data-independent acquisition technique which produces MS/MS like information on all precursor ions where fragment ions are correlated to their respective precursors through a frequency of fragmentation induced by modulating precursor ions through a fragmentation zone at a modulation frequency corresponding to an ion’s *m*/*z*. The most developed variation of 2DMS is the original technique developed in the 1980s.^39,41,42^ Ions are irradiated with a specific sequence of radio frequency sweeps, known hereafter as *the* Gäumann pulse sequence after one of the original researchers. In the Gäumann pulse sequence, ions are excited first by a low-energy frequency sweep which excites them into a higher orbit. There is then a delay (*t*_1_) that allows the ions to precess around the cell at an angular momentum proportional to their cyclotron frequency. The delay allows the ions to gain a phase prior to the second low-energy excite pulse, which in turn excites or de-excites the ions dependent on their phase with respect to the second excite pulse. The ions are then subjected to a radially dependent fragmentation technique, in this case a CO_2_ laser (Synrad, Uk) performing infrared multiphoton dissociation (IRMPD). There is then a standard excite and detect process, and the pulse sequence is repeated across many scans incrementally increasing *t*_1_ and thereby the phase lag for each ion between the first and second excitation pulse and causing them to periodically be inside or outside of the fragmentation zone at the center of the cell. Over the set of experiments, the frequency of the modulation in precursor ion intensity will equal the frequency of modulation of fragment ion intensity with opposite phases, and so fragments can be correlated to precursors using the Fourier transform in the precursor ion (modulation) dimension which is done in 2DMS processing.

While the Gäumann pulse sequence is limited to FTICR MS, other methods of 2DMS exist, including using stored waveform ion modulation,^43,44^ and more recently it was shown that any reproducible and calibratable separation of ions can be exploited to perform 2DMS correlations.^45^

2DMS has been widely used in the analysis of complex bio mixtures,^40,46–50^ polymers,^51^ and small molecules.^52–54^ The everything-all-at-once nature of a 2DMS experiment means that every component of the spectrum that fragments with the given fragmentation technique will be present within the 2DMS spectrum, which leads to a reduction of bias within the acquisition. However, the size of the data set makes data handling, processing, and analysis challenging.

In 2DMS the precursor *m*/*z* is generally plotted along the *y* axis (vertical) with fragment *m*/*z* plotted along the *x* axis (horizontal), which means that common fragments of different precursors can be found through extraction of the vertical axis at a given fragment *m*/*z*, and MS/MS like fragmentation spectra can be created by extracting the horizontal axis at a given precursor *m*/*z*. If there is a common neutral loss for different precursors in the sample, it will appear as a line parallel to the autocorrelation line. There are other techniques such as 2D MS/MS^55–58^ and 2D autocorellation mass spectrometry^59^ which show similar data differently.^60,61^

**Figure 1:**
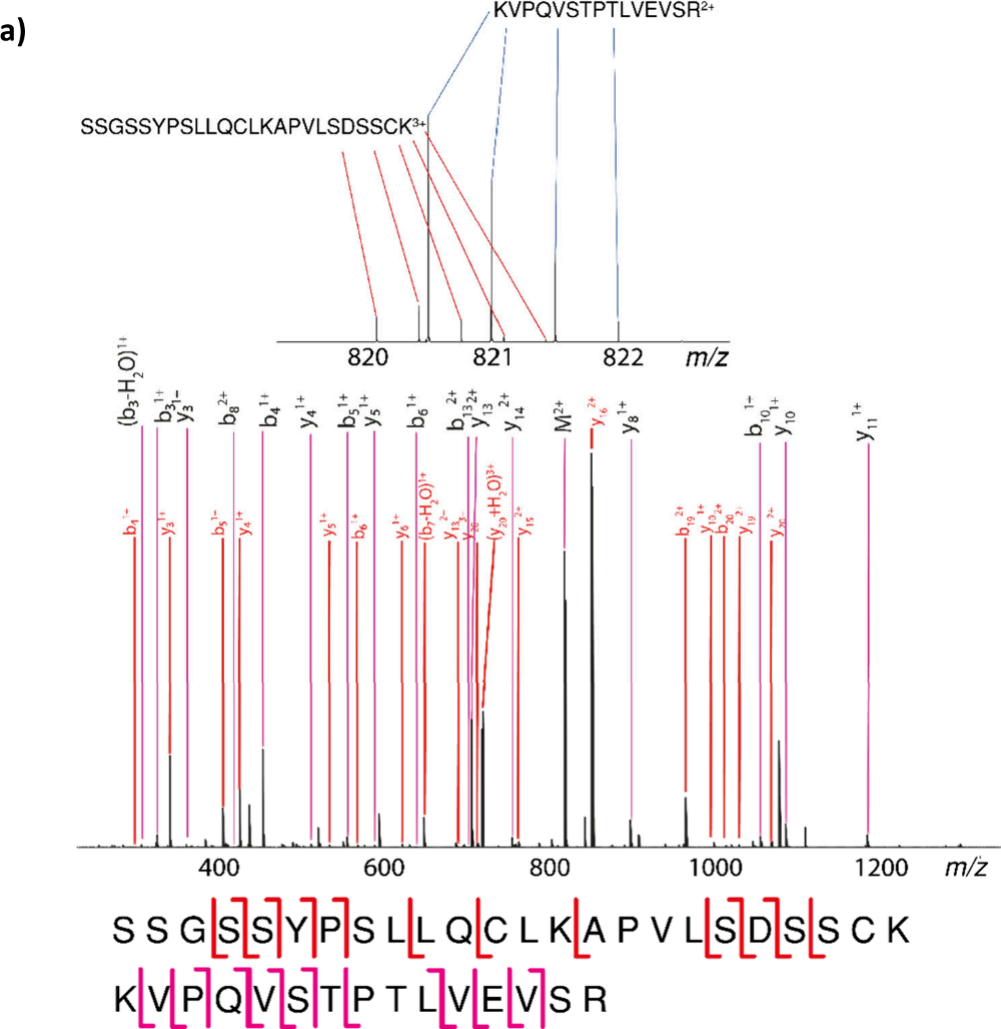
(a) Zoomed-in view of quadrupole isolated BSA peptide. (b) MS/MS spectra exhibiting chimeric character due to a coisolated tryptic autodigest species.

2DMS has a huge peak capacity as it maps the MS precursor and fragment ion data in two dimensions. The peak capacity (PC) can be found by multiplying the peak capacity in the precursor dimension by the peak capacity in the fragment dimension. One way to define the theoretical maximum peak capacity is defined as

$$\mathrm{theoretical peak capacity}=\frac{\mathrm{mass range}\left(P\right)}{\mathrm{fwhm}\left(P\right)}\times \frac{\mathrm{mass range}\left(F\right)}{\mathrm{fwhm}\left(F\right)}$$ 8

where the theoretical peak capacity is a measure of the maximum number of resolved peaks within the spectrum; fwhm is the full width at half-maximum resolution; mass range is the accepted mass range; and F and P are fragment and precursor axes, respectively. In the case of 2DMS on an FTICR, the peak capacity can be easily in the range of hundreds of millions.

Peak capacity values for TIMS are generally defined by the number of slices taken where the slices taken are larger than the resolving power. However, mathematically, peak capacity (PC) can be related to resolving power, *R*, through the equation

PC=R×ramp scan width 9

The maximum peak capacity for TIMS is usually in the hundreds for TIMS-FTICR; however, it is important to note that ion mobility is only semi orthogonal to *m*/*z*, and that not all of the mobility-*m*/*z* space is occupied; in addition, not all of the *m*/*z* nor the mobility space is usually occupied; therefore, theoretical PC values will always be larger than the actual PC. If TIMS were to be combined with FTICR 2DMS these PC values would be multiplied once again as the data set would now be a 3D coordinate space with a fourth dimension of intensity. Therefore, the theoretical peak capacity of a TIMS-2DMS experiment could be upward of tens of billions, which allows for the analysis of extremely complex mixtures; however, there may be limitations in the possible assignable space due to not all of the mobility space or the mass domain being populated and due to the inherent ion capacity of the instrument multiplied by the number of individual mass spectral scan lines. Ion mobility hyphenation also allows the possibility of MS/2DMS on a species within a complex mixture where near *m*/*z* species may make it difficult to isolate individual species with traditional quadrupole methods.^52–54^

MS/2DMS functions similarly to MS^3^ in that it produces a map of fragments from the fragment precursors that they are generated from. In large-chain molecules such as proteins or polymers it is common for there to be a deficiency in the fragmentation efficiency of the core region of the chain as compared to the fragmentation efficiency of the outer regions of the chain. Higher-energy dissociation may cause ions already fragmented to fragment again causing secondary fragments.^62^ It is possible to assign labels to these species using statistical methods such as prediction of likely secondary fragmentation, or correlating the intensity of the ions which may generate the fragments.^62,63^ However, using MS/2DMS or MS^3^ there is no need for statistical methods as the secondary fragments are correlated to their primary fragments via the modulation frequency, which removes the ambiguity as to which secondary fragment came from which primary fragment.

## Methods

### Chemicals

Bovine serum albumin (BSA) (A2153), melittin from honey bee venom (M2272), ubiquitin from bovine erythrocytes (U6253), carbonic anhydrases 2 from human (CAII)­(C6165), human insulin (I2643), lysozyme from chicken egg white (L6876), and human hemoglobin (H7379) were purchased from Merck.

For the bottom-up analysis, the proteins were digested by first reducing with dithiothreiol (DTT) (D-9779) and alkylating with iodoacetamide (IAA) (11149), followed by digestion with trypsin (V5111 from Promega). 6 μL of DTT (6 μL, 8 mg/mL) suspended in 100 mM ammonium bicarbonate (ABC) (09830) was added to 0.1 mg of BSA in 0.2 mL of 100 mM ABC and shaken at 1400 rpm and 60 °C using the Eppendorf Thermomixer C (Eppendorf Uk) for 30 min. 6 μL of IAA (19 mg/mL in 100 mM ABC) was then added to the resultant mixture, and the mixture was left in the dark for 60 min, after which 4 μL of trypsin (1 mg/mL in 100 mM ABC) was added; the mixture was then shaken at 37 °C for 16 h using a thermomixer (Eppendorf Thermomixer C). The digested protein was then collected using a sola μ C18 spin down column from Thermo-Fisher Scientific.

In order to examine protein mixtures, BSA digest was mixed with intact melittin, ubiquitin, insulin, lysozyme, and hemoglobin at a 1:1:1:1:1:1 ratio.

### Instrumentation

All experiments took place on a modified 12 T solariX FTICR mass spectrometer (Bruker Daltonics, Germany). Ionization was accomplished through a home-built nano-ESI (n-ESI) source set up as the following: a thin glass capillary (TW120F) (World Precision instruments WPI, UK) was pulled to a fine tip with a Sutter p97 flaming brown tip puller (World Precision Instruments WPI); the analyte solution was then loaded into the tip. and the tip was grounded though an internally placed nichrome wire (Jacobs Online, USA). The tip was then held at 800 V near a spray shield at the instrument entrance to enable nano-ESI to occur. The 12 T FTICR at the University of Warwick has been upgraded to include a gated TIMS cartridge, by Dr. Chris Wootton of Bruker, which allows for experiments using gated TIMS, similar to the approach in refs 45 and 46 . For calibration purposes the gate was set to a 3 V width and swept from −200 V to −30 V in steps of 0.5 V.

### 2DMS Parameters

In order to perform the 2DMS experiment, a previously described pulse program was used known as the Gäumann pulse sequence. The individual FTICR mass spectra acquired within this 2DMS experiment were 2 MPoint (2^21^ data points stored as 32-bit integers) in length, and the 2DMS experiment consisted of 2048 individual FTICR mass spectra. The low *m*/*z* of each scan was 147.09428 (about 1.25 MHz at 12 T), and the 2DMS T1 delay increment was 1.1 μs per scan.

The spectra were processed using Touchstone 2DMS, an in-house software suite which performs the two fast Fourier transforms (FFTs) as well as zero-filling and apodization. A 3D peak list was generated using T2D,^46,53^ another in-house software which allows for 3D peak-picking of 2DMS data, which was used to identify fragments along the autocorrelation line and secondary fragments along horizontals from those fragments. From these assignments, it was possible to characterize each TIMS 2DMS experiment individually and quickly.

## Results and Discussion

One major benefit of using an ion mobility separation over an *m*/*z*-based separation is that the mobility axis is semiorthogonal to *m*/*z* in that both ion mobility and *m*/*z* share a charge component. Size does not scale equally with mass across all molecules, and therefore, molecules that are very close in mass may be separated based on size, which means that near *m*/*z* species, or even isomeric or isonucleonic^64^ species, can be separated using TIMS. Near *m*/*z* species can cause chimeric MS/MS spectra, meaning that multiple species fragments are present in the same spectra. An example of near *m*/*z* species was found near a BSA peptide (KVP­QVS­TPT­LVE­VSR) as shown in Figure 1. The sample contains several peptides from tryptic autodigest (when the digestion enzyme trypsin digests itself). Figure 1a shows an instance in which the isotope pattern of a tryptic autodigest species overlaps with the isotope pattern of a BSA peptide. Many of the high-intensity peaks within the spectrum belong to the trypsin peptide, and so if the nature of the peptide was unknown, the spectra would be incredibly challenging to assign.

While the two peptides exhibit overlapped isotope distributions, they are distant in mobility space which means that ion mobility can be used to fully isolate the intended peptide without the additional tryptic autodigest species. The isolated peptide can then be fragmented and assigned without the chimeric species, as shown in Figure 2.

**Figure 2:**
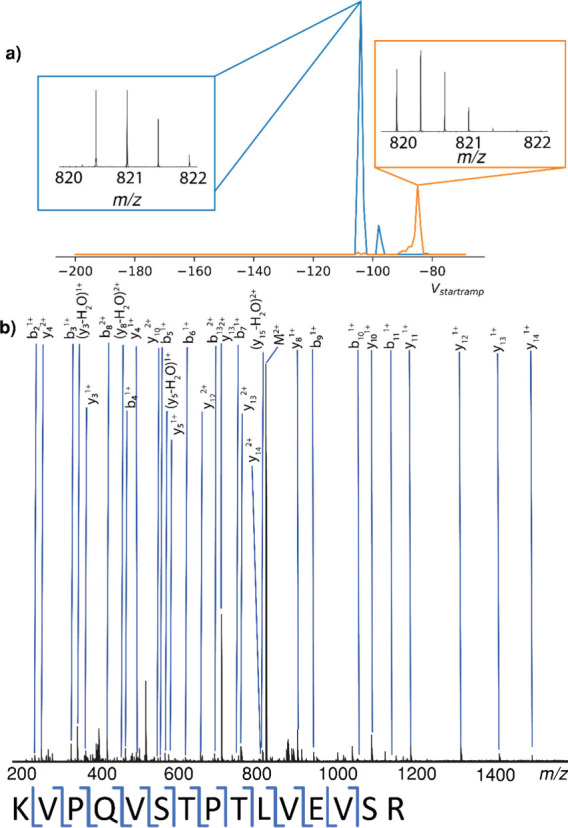
(a) Extracted ion mobilograms of the near *m*/*z* species of interest at *m*/*z* 820 inf BSA digest showing clear separation of the BSA peptide from the tryptic peptide. (b) TIMS isolated BSA peptide CID MS/MS spectrum showing less chimeric character.

In order to determine the separating efficiency of the technique, two “near *m*/*z*” species were identified, which are the 9+ charge state of ubiquitin (around *m*/*z* 952) and the 3+ charge state of melittin (around *m*/*z* 949). While the separation of the monoisotopic peaks are possible with a quadrupole, albeit with signal losses, separation of later isotopes of melittin can be challenging to a traditional system and coisolation would be a concern. Using trapped ion mobility, it was possible to separate these two species easily with the 9+ Ubiquitin peak eluting at −127 V_ramp start_ and the 3+ melittin peak eluting at 134 V_ramp start_ as shown in Figure 3d. After isolation, it was possible to perform collision-induced dissociation (CID) of the melittin 3+ species without bleed through from the 9+ ubiquitin species, which can be seen in Figure 4.

**Figure 3:**
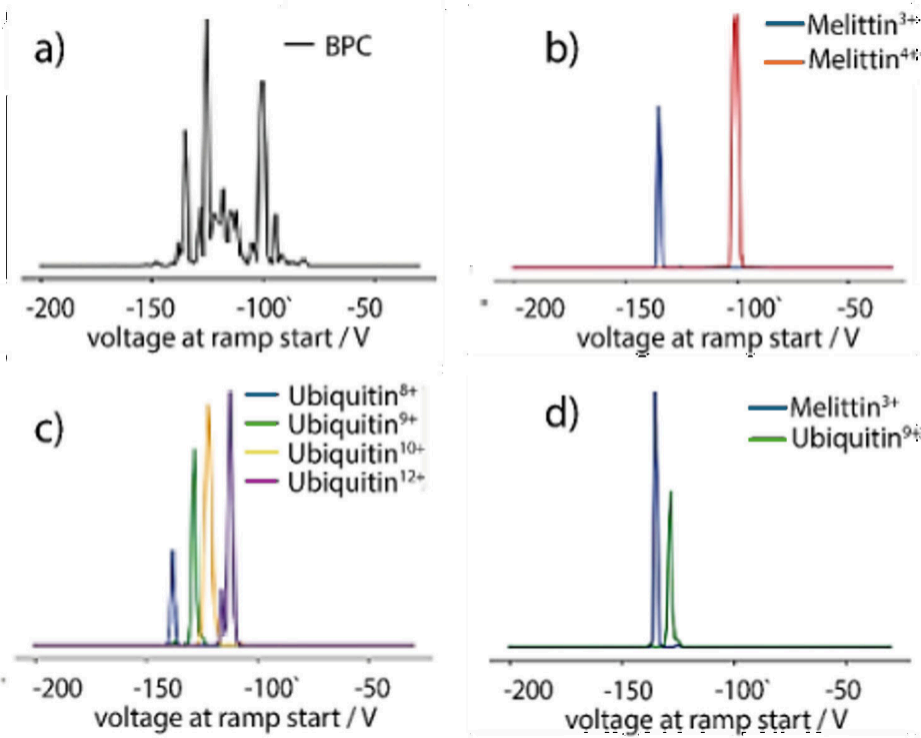
(a) TIMS spectra of a mixture of ubiquitin and melittin, (b) extracted ion mobiligrams of melittin charge states, (b) extracted ion mobiligrams of ubiquitin charge states, and (d) separation of near *m*/*z* species of melittin and ubiquitin.

**Figure 4:**
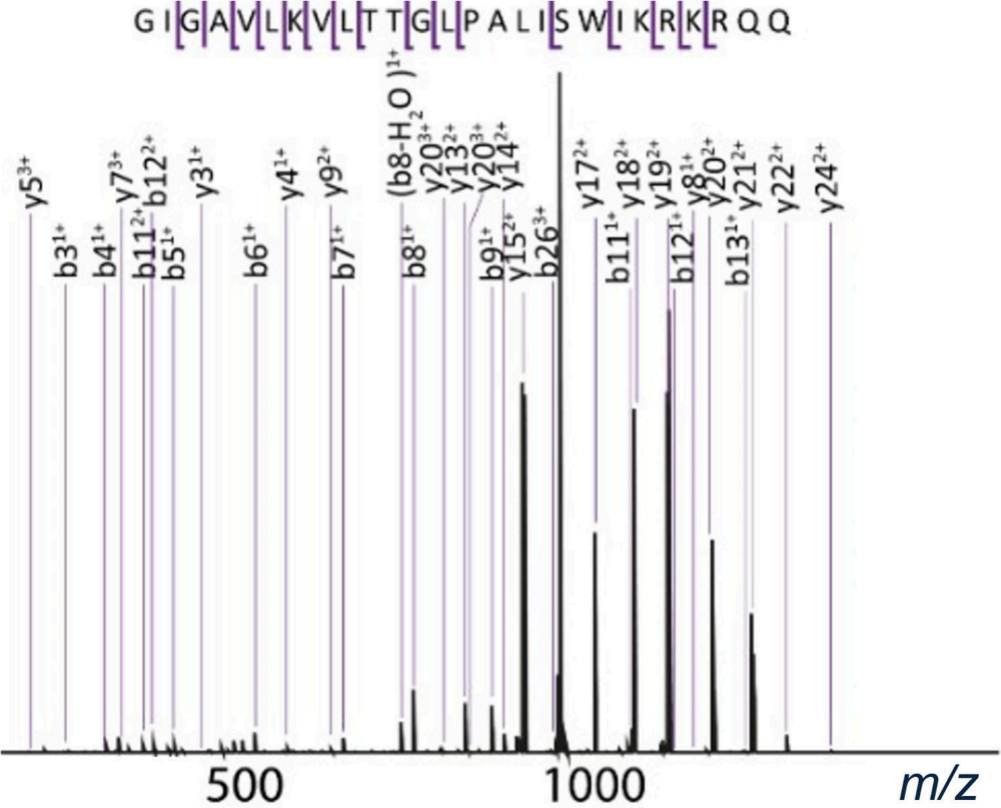
TIMS isolated MS/MS spectra of the 3^+^ charge state of melittin isolated at *m*/*z* 949.

In the CID spectrum of melittin, shown in Figure 4, there were some low-intensity peaks that could correspond to secondary fragments (fragments of fragments). Generally these peaks either go unassigned, or statistical methods have to be used in order to determine which fragment each secondary fragment came from and thus the validity of the assignment. Using MS/2DMS, there is less ambiguity around which fragment each secondary fragment came from as secondary fragments are correlated to their precursor fragment via their modulation frequencies. In order to perform MS/2DMS, melittin was isolated from the mixture of proteins and peptides and CID was performed. After CID, a 2DMS of these primary fragments using IRMPD was taken with the parameters in the Methods section.

The MS/2DMS style of experiment^50,65^ allows for the analysis of internal fragments, which is particularly useful in the analysis of the central regions of a protein which may not be available through traditional methods.

As can be seen in Figure 5, due to the correlation of secondary fragments with their precursor primary fragment, it is possible to assign secondary fragments with great confidence. In Figure 5A, the primary fragment spectrum can be extracted along the autocorrelation line (the *x* = *y* diagonal) to find all of the fragment ions from the initial CID fragmentation of the 3+ ion of melittin (observed at around *m*/*z* 949.59) and projected to form a scan of the 2DMS precursor ions. Figure 5C is the 2DMS surface; two horizontal slices of the surface were extracted at *m*/*z* 1104.18, the y_19_^2+^ primary fragment ion, to form the spectral projection in Figure 5B and at *m*/*z* 812.4940, the [M+3H]^3+^ ion, to form the spectral projection in Figure 5D, which shows that the additional fragmentation is not simply due to the different fragmentation technique.

**Figure 5:**
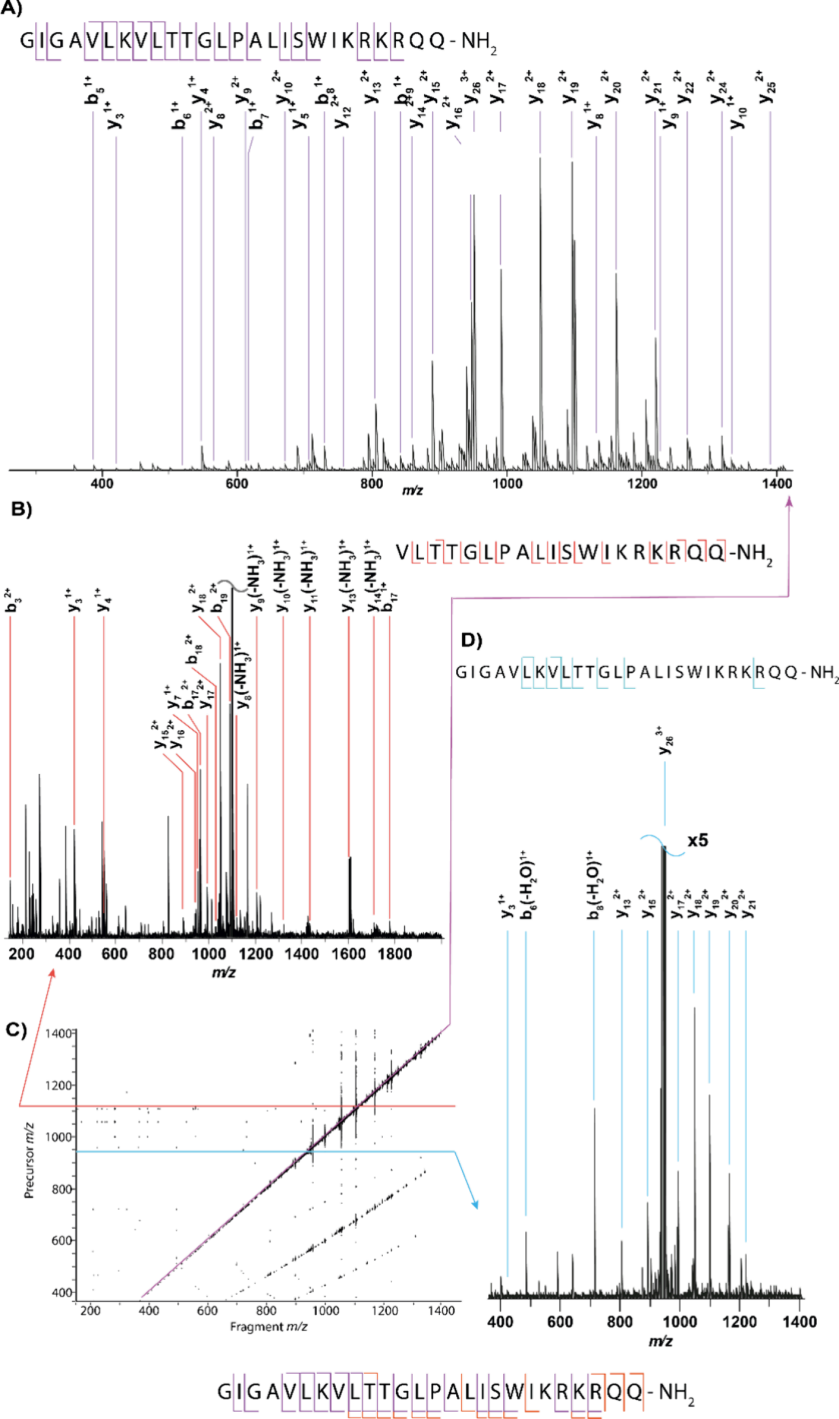
(A) Autocorrelation line of a TIMS-MS/2DMS experiment on melittin, (B) extracted horizontal line demonstrating the ability to identify secondary fragmentation, (C) overview contour plot of the 2DMS experiment, and (D) extracted horizontal line of intact melittin.

By extracting the autocorrelation line, it is possible to assign the primary fragments resulting from CID in the front end, in which 73% cleavage coverage was achieved, which is an improvement over the 61% achieved in traditional MS/MS shown above. 2DMS takes advantage of the Fellgett signal averaging effect. In 2DMS many scans must be taken (2048 in Figure 5) and each scan is a consecutive sampling point in the time dimension; therefore, the signal-to-noise will increase with the square root of the number of scans.^66^ As a result, low-intensity peaks that may be lost in the noise of typical MS/MS will appear with more prominence in 2DMS.

It was possible through assignments of secondary fragments to increase sequence coverage to 92% which allows for deeper analysis of proteins in situ. It may also be possible to use the precursor dimension (vertical in 5C) to determine if some fragments are shared within multiple precursors, as might be expected for secondary fragmentation, as multiple fragmentation routes can lead to the same fragment. However, this is complicated by the high noise in vertical scans which comes from differences in scan-to-scan intensity of ion signal separate from factors within the 2DMS experiment (differences in ion current, shot-for-shot fragmentation frequency, etc.).^45^

It is worth noting that the near *m*/*z* species studied were resolved fully in the ion mobility dimension. If this were not the case and the near *m*/*z* species were only partially resolved, the analysis may prove more challenging; it is possible that using multi-dimensional peak picking techniques may make the analysis in this case possible; however, this remains a challenging problem. Another solution could be found in the profile spectrum of the mobility space: as there is often some width associated with a peak, it may be possible to sacrifice intensity of a precursor ion to gain resolution from a near *m*/*z* neighbor.

## Conclusions

Using trapped ion mobility spectrometry it is possible to separate out large biomolecules from near *m*/*z* neighbors for deeper characterization. Using MS/2DMS it was possible to get a much deeper cleavage coverage of the top-down protein melittin through confident identification of secondary fragmentation by using the ability of 2DMS to distinguish fragments based on their respective precursor. Therefore, it is possible to confidently assign secondary fragments directly to the primary fragment of origin, allowing for less ambiguity in the assignment of secondary fragments within the spectrum.
